# Antimicrobial Resistance and Wildlife: Occurrence of Antimicrobial Resistance Genes in Red Foxes (*Vulpes vulpes*, Linnaeus, 1758), in Italy

**DOI:** 10.3390/ani15142022

**Published:** 2025-07-09

**Authors:** Antonietta Di Francesco, Daniela Salvatore, Roberta Taddei, Fabrizio Bertelloni, Caterina Lupini, Giulia Cagnoli, Valentina Virginia Ebani

**Affiliations:** 1 Department of Veterinary Medical Sciences (DIMEVET), University of Bologna, 40064 Bologna, Italy; daniela.salvatore2@unibo.it (D.S.); caterina.lupini@unibo.it (C.L.); 2 Istituto Zooprofilattico Sperimentale della Lombardia e dell’Emilia Romagna (IZSLER), “Bruno Ubertini”, Sede Territoriale di Bologna, 40127 Bologna, Italy; roberta.taddei@izsler.it; 3 Department of Veterinary Sciences, University of Pisa, 56124 Pisa, Italy; fabrizio.bertelloni@unipi.it (F.B.); giulia.cagnoli@phd.unipi.it (G.C.); valentina.virginia.ebani@unipi.it (V.V.E.)

**Keywords:** antimicrobial resistance, antimicrobial resistance genes, wildlife, red fox, *Vulpes vulpes*, PCR, Italy

## Abstract

Clinically significant antimicrobial-resistant bacteria and resistance genes are increasingly being reported in wildlife. In this study, 127 splenic samples from red foxes (*Vulpes vulpes*) from northern and central Italy were analysed for the presence of resistance genes against antimicrobials such as tetracycline, sulphonamide, β-lactam, and colistin, which were previously extensively used in human and veterinary management of bacterial diseases. One or more antimicrobial resistance genes were detected in 78 (61%) of 127 splenic samples. Polymerase chain reaction positivity was revealed for 13 genes—*tet*(A), *tet*(B), *tet*(K), *tet*(L), *tet*(M), *tet*(O), *tetA*(P), *tet*(Q), *tet*(S), *tet*(X), *sul1*, *sul2*, and *bla*_TEM-1_—out of the 21 tested genes. Our results, corroborated by reports in the literature, confirm the potential role of the red fox as a sentinel for antimicrobial-resistant bacteria in contaminated environments and suggest that detecting resistance genes in biological samples by a culture-independent method might be an effective tool for the epidemiological study of antimicrobial resistance in wildlife.

## Simple Summary

Antimicrobial resistance is a widespread problem, occurring in clinical, veterinary, agricultural, and environmental settings, strongly influenced by human activities like the overuse and misuse of antimicrobials. In this study, an epidemiological investigation of the spread of antimicrobial resistance genes was performed by testing splenic samples of 127 red foxes via a molecular approach. Positivity for one or more resistance genes was found in 78 (61%) of the samples tested. These results confirm that foxes, which feed on human waste, scavenge contaminated carcasses, and consume peri-domestic prey, could be sentinels of the presence of antimicrobial resistant bacteria in contaminated environments.

## 1. Introduction

Antimicrobial resistance (AMR) is considered a critical global health challenge that spans all domains of the One Health framework. In the veterinary field, most studies on AMR have focused on intensively reared cattle, pigs, poultry, and fish because these animals are more frequently exposed to pharmacological pressure. However, from a One Health perspective—recognising the interconnectedness of humans, animals, and the environment—wildlife is an integral part of the environment [1] and cannot be excluded from AMR investigations. AMR in the environment can occur naturally [2], but it is also significantly influenced by anthropogenic pressures [3]. Wildlife clearly demonstrates how such human pressures can increase and accelerate the development and spread of AMR [4]. Wild animals are generally less exposed to pharmacological treatments than domestic animals and humans [5], except in specific cases, such as those treated in wildlife recovery centres. Nevertheless, human population growth and land-use changes, including rapid urbanisation, have resulted in the fragmentation or loss of natural habitats available to wild animals [6], thus eroding natural barriers between humans, domestic and domesticated animals, and wildlife. As a result, natural ecosystems are increasingly subject to anthropogenic pressures, which, in turn, affect the resistome of wild animals through environmental contamination via wastewater or manure [7]. Clinically significant antimicrobial-resistant bacteria and AMR genes (ARGs) are increasingly being reported in wildlife, including red foxes [8,9,10,11,12,13,14,15,16], although the real implications of AMR for wildlife health remain unclear [17]. In most cases, these studies have relied on bacteriological culture, antibiotic susceptibility testing of isolated microorganisms, and subsequent molecular identification of ARGs.

The purpose of this study was to investigate, using a culture-independent molecular approach, splenic samples from red foxes from northern and central Italy for the presence of genes that confer resistance to antimicrobials which were extensively used in both human and veterinary medicine before the current restrictions in force in the EU, especially in the animal sector.

## 2. Materials and Methods

### 2.1. Sampling

Total DNA was previously extracted from splenic samples of 127 red foxes (*Vulpes vulpes*, Limnaeus, 1758) culled as part of wildlife population control plans between 2022 and 2024. Ninety-eight foxes were sent to the Department of Veterinary Sciences at the University of Pisa in central Italy for teaching purposes, and 29 were sent to the Istituto Zooprofilattico Sperimentale Lombardia and Emilia-Romagna (Sede Territoriale di Bologna) in northeastern Italy for diagnostic investigations. Splenic samples were collected during post-mortem examinations carried out within 24 h of death and stored at −20 °C until DNA extraction.

DNA extraction was performed using the QIAamp DNA Mini Kit (Qiagen, Hilden, Germany), following the manufacturer’s instructions. Negative controls (kit reagents only) were included in each extraction set. The DNA extracts were stored at −20 °C and subsequently sent to the Department of Veterinary Medical Sciences at the University of Bologna, Italy, for molecular assays.

### 2.2. Molecular Analysis

PCR was performed by targeting the following ARGs: *tet*(A), *tet*(B), *tet*(C), *tet*(D), *tet*(E), *tet*(G), *tet*(K), *tet*(L), *tet*(M), *tet*(O), *tet*(S), *tetA*(P), *tet*(Q), *tet*(X), *sul1*, *sul2*, *sul3*, *bla*_CTX-M_, *bla*_SHV_, *bla*_TEM_, and *mcr*-1.

Each gene was amplified by an individual PCR, using primers according to Ng et al. [18] (*tet* genes), Sáenz et al. [19] (*sul* genes), Batchelor et al. [20] (*bla*_CTX−M_), Jouini et al. [21] (*bla*_SHV_ and *bla*_TEM_), and Liu et al. [22] (*mcr*-1). The following PCR protocols were applied: initial denaturation at 94 °C for 5 min, followed by 35 cycles at 94 °C for 1 min; annealing at 50 °C [*tet*(K)], 51 °C [*tetA*(P), *tet*(S), and *sul3*], 53 °C [*tet*(B), *tet*(D), *tet*(E), *tet*(M), *tet*(Q), *tet*(X), *bla*_SHV_, and *mcr*-1], 55 °C [*tet*(A), *tet*(C), *tet*(G), *tet*(L), *tet*(O), *sul2*, *bla*_CTX-M_, and *bla*_TEM_], or 59 °C (*sul1*), each for 1 min; and extension at 72 °C for 1 min. A final extension step at 72 °C for 10 min completed the reaction. DNA from *Escherichia coli* strains containing AMR plasmids was used as a positive control.

The PCR products were analysed by 2% agarose gel electrophoresis; DNA bands were stained with Midori Green Advance (Nippon Genetics Europe GmbH, Düren, Germany) and visualised using ultraviolet trans-illumination. All amplicons were purified using the NucleoSpin Gel & PCR Clean-up Mini Kit (Makerey-Nagel, Duren, Germany), and both DNA strands were sequenced using the Sanger method (Bio-Fab Research, Rome, Italy). The resulting sequences were edited and assembled using Bioedit software v7.7 and compared with publicly available sequences via the nucleotide BLAST (v2.16.0) server in the GenBank database (National Center for Biotechnology Information 2025).

### 2.3. Statistical Analysis

The chi-square (χ^2^) test was used to compare the percentage of ARG-positive samples between northern and central Italy. The threshold for statistical significance was set at *p* ≤ 0.05.

## 3. Results

The results are shown in Table 1.

In total, 78 (61%) of the 127 splenic samples contained one or more ARGs. The positivity rates for the two regional groups of animals tested, based on their origin, were similar: 62% for samples from central Italy and 59% for samples from northern Italy. Statistical analysis revealed no significant differences (*p* > 0.05) in the positivity rates between the northern and central Italy samples.

Thirteen of the 21 tested genes were detected. Specifically, amplicons for *tet*(A), *tet*(B), *tet*(K), *tet*(L), *tet*(M), *tet*(O), *tetA*(P), *tet*(Q), *tet*(S), *tet*(X), *sul1*, *sul2*, and *bla*_TEM-1_ were observed, whereas no positive results were obtained for *tet*(C), *tet*(D), *tet*(E), *tet*(G), *sul3*, *bla*_CTX-M_, *bla*_SHV_, or *mcr*-1.

The *tet*(M) gene was detected in 31% of the samples, followed by *tet*(O) in 26% and *tetA*(P) in 21%.

For each amplified gene, the identity of the amplicons was confirmed by comparing the obtained sequences with corresponding entries in the GenBank database, showing 99–100% nucleotide similarity. One sequence for each of the 13 detected genes was deposited in the GenBank database under accession numbers PV299117–PV299129.

## 4. Discussion

The red fox is considered the medium-sized canid with the widest global distribution [23], and it is one of the most successful infiltrators of urbanised environments. A key factor in the fox’s adaptability is its highly flexible diet, which includes invertebrates (insects, earthworms), amphibians, reptiles, small mammals, birds and their eggs, fruits and berries in spring and summer, and food sources found in landfills. The ecological plasticity of this opportunistic predator enables it to adapt well to rural, urban, and peri-urban environments, making it a potential sentinel in various fields of study. These include the detection of environmental contaminants such as organochlorine pesticides or heavy metals [24], the occurrence and spread of zoonotic diseases, and the effects of climatic change on habitats [25]. Furthermore, foxes that feed on human waste, scavenge contaminated carcasses, or consume peri-domestic prey can serve as bioindicators of AMR in the environment. In this context, Mo et al. [12] reported that foxes living in urban areas of Norway are more likely to be exposed to antimicrobial-resistant bacteria than foxes living in remote areas, confirming the decisive role of anthropogenic pressure in the development of AMR in foxes.

Although it is very difficult to ascertain the exact size of the fox population because of the species’ elusive nature and primarily nocturnal habits, *V. vulpes* is believed to be widely distributed throughout Italy and does not face any specific conservation issues, despite being regularly hunted and subjected to numerical control measures [26].

Previous studies performed in Italy have described the occurrence of bacterial pathogens in faecal matter or intestinal contents from red foxes [10,27,28,29,30,31,32,33,34,35], but only a few of these studies [10,28,30,33] examined the antimicrobial susceptibility of the isolated bacterial strains.

In our study, DNA extracted from splenic samples of red foxes was tested for genes encoding resistance to tetracycline, sulphonamide, β-lactam, and colistin antimicrobials. One or more ARGs were detected in 61% of the splenic samples. This high percentage of positivity may be linked to the fact that both areas of origin are densely populated by humans, suggesting significant anthropogenic pressure on the fox populations.

The detection of ARGs in the splenic samples suggests their association with bacteria responsible for systemic infection related to bacteraemia. Spleen samples are commonly used as samples for detecting pathogens due to the spleen’s role in filtrating blood. The negativity of the samples tested in this study does not necessarily coincide with the absence of infectious bacteria but exclusively indicates the absence of bacteria carrying the genes being investigated. Cross-contamination with intestinal contents can be excluded because the DNA was extracted from intact spleens.

The highest positivity was observed for *tet* genes, detected in 55% of the splenic samples tested. This aligns with the widespread phenomenon of tetracycline resistance [36], which results from its intensive use in treating human and animal infections due to the antimicrobial’s broad-spectrum activity, low cost, and low toxicity. In our study, we tested 14 *tet* genes involved in the three known tetracycline resistance mechanisms: tetracycline efflux pumping [*tet*(A), *tet*(B), *tet*(C), *tet*(D), *tet*(E), *tet*(G), *tet*(K), *tet*(L), and *tetA*(P)], ribosomal protection [*tet*(M), *tet*(O), *tet*(Q), and *tet*(S)], and enzymatic inactivation [*tet*(X)]. The high frequency of the *tet*(M) gene, detected in 31% of the samples, is not surprising because this gene has been identified in at least 82 bacterial genera [37,38]. This is likely because of its association with conjugative transposons, which appear to have lower host specificity than plasmids [39].

The broad-spectrum bacteriostatic activity of sulphonamides accounts for their widespread use in both human and veterinary medicine worldwide [40], leading to high prevalence rates of sulphonamide resistance, particularly in Gram-negative bacteria from animals and humans [41]. The target of sulphonamides is the enzyme dihydropteroate synthase in the folic acid pathway. Sulphonamide resistance is plasmid-borne and associated with the presence of *sul* genes, which encode a variant of the dihydropteroate synthase enzyme with low affinity for sulphonamides. In our study, *sul* genes were detected in 24 (19%) of the 127 samples tested—specifically *sul1* in 13% and *sul2* in 6%. No positive results were obtained for the *sul3* gene, consistent with studies in the literature reporting a higher frequency of *sul1* and *sul2* genes [42].

β-lactam antibiotics are among the most commonly used antibiotics because of their minimal side effects and broad antibacterial spectrum. The major mechanism of β-lactam resistance, particularly among Gram-negative bacteria, is the production of β-lactamases—hydrolytic enzymes that break down the β-lactam ring, rendering the antibiotic ineffective. Resistance to β-lactams is increasing, posing a public health concern worsened by the rapid evolution of extended-spectrum β-lactamases (ESBLs), a group of enzymes that confer resistance to most β-lactam antibiotics, including expanded-spectrum cephalosporins and monobactams [43]. The most common types of ESBLs are variants of the CTX-M, SHV, and TEM enzymes, resulting from amino acid substitutions that alter their substrate profile. In our study, 8% of the samples contained the *bla*_TEM-1_ gene, which encodes the TEM-1 β-lactamase. This gene requires only a few specific single-nucleotide polymorphisms to evolve into a gene encoding an ESBL [44].

Our results, supported by reports in the literature, confirm the potential role of the red fox as a sentinel for AMR in contaminated environments [12]. Furthermore, the detection of ARGs in biological samples using a culture-independent method—although it does not allow for identification of the bacterial species involved—seems to be an effective tool for epidemiological research. This approach helps avoid potential underestimation of AMR due to non-culturable or slow-growing bacteria [45], as well as the advanced decomposition of carcasses, which often occurs in wildlife studies [46,47,48,49].

Despite the growing number of reports on AMR in wildlife, many aspects related to the potential risk posed by wild animals to public and animal health remain unclear, as do the real implications of AMR for wildlife health itself [17]. Currently, the role of wildlife as a reservoir or vector for the spread of AMR remains hypothetical and could be better understood through active surveillance. This could include comparing bacteria isolated from wildlife with those from humans or domestic animals, as well as conducting longitudinal sampling over extended periods to assess the persistence and dissemination of AMR by wildlife within and between regions [50,51]. Unfortunately, within current government strategies, most attention is focused on AMR surveillance in humans, domestic and domesticated animals. Few countries mention wildlife in their AMR national action plans, and only a small number have implemented an operational phase for wildlife surveillance [17].

## 5. Conclusions

Antimicrobial resistance in natural ecosystems is of increasing concern. Many studies have documented the presence of AMR in wildlife, proposing constant, standardised monitoring of AMR occurrence in wildlife to support a better understanding of the One Health dimension of AMR. The results of this study agree with previous reports suggesting a role of wildlife as sentinels of the presence of resistant bacteria and/or genetic determinants of antimicrobial resistance in the environment.

## Figures and Tables

**Table 1:** Antimicrobial resistance genes detected in splenic samples from red foxes in Italy. ID = sample identification number; ARGs = antimicrobial resistance genes. Samples from central Italy are numbered 1–98; samples from northern Italy are numbered 99–127.

ID	ARGs	ID	ARGs	ID	ARGs	ID	ARGs	ID	ARGs	ID	ARGs
3	*tet*(M), *tet*(O)	28	*tet*(A), *tet*(B), *tetA*(P), *sul1*, *sul2*, *bla*_TEM-1_	47	*tetA*(P)	63	*tet*(M), *tet*(O), *tetA*(P)	91	*tet*(M), *tetA*(P)	111	*sul1*
4	*tetA*(P), *sul1*	29	*sul1*, *bla*_TEM-1_	49	*tetA*(P)	64	*tet*(L), *tet*(M), *tet*(S)	95	*tet*(A), *tet*(L), *tet*(M), *tetA*(P), *tet*(S)	113	*tet*(O)
5	*tet*(O)	33	*tet*(O)	50	*tet*(O)	65	*tet*(B), *tet*(M), *tet*(S), *sul1*, *bla*_TEM-1_	96	*tet*(M), *tet*(O)	114	*tet*(B), *tet*(L), *tet*(M), *tet*(X), *bla*_TEM-1_
6	*tet*(M)	34	*tetA*(P), *sul1*	51	*sul1*	66	*tet*(M), *tetA*(P), *tet*(S), *sul1*	97	*tet*(L), *tet*(M), *tet*(O)	117	*tet*(O), *tet*(S)
7	*tet*(O), *tetA*(P)	36	*tet*(O)	52	*tet*(O)	67	*tetA*(P)	98	*tet*(L), *tet*(M), *tet*(O)	118	*tet*(B), *tet*(L), *tet*(M), *tet*(S), *tet*(X), *sul1*
9	*tet*(O), *sul1*	37	*sul2*, *bla*_TEM-1_	53	*tet*(O), *tet*(Q), *tet*(X)	68	*tet*(B), *tet*(M), *tetA*(P), *sul1*	99	*tet*(L), *tet*(M), *tet*(O), *bla*_TEM-1_	120	*tet*(X)
10	*tet*(O)	38	*tet*(A), *tet*(B), *tet*(M), *tetA*(P), *sul1*, *sul2*, *bla*_TEM-1_	55	*tet*(K), *tet*(L), *tet*(M), *tet*(O), *tet*(Q), *tet*(X), *sul2*	75	*tet*(M)	100	*tet*(X)	122	*tet*(M)
12	*sul1*	39	*tet*(M)	56	*tet*(M), *tetA*(P)	76	*tet*(L), *tet*(O)	101	*tet*(M), *tet*(O), *tet*(X), *sul2*	123	*tet*(B)
13	*tet*(M), *tet*(O), *tet*(X)	40	*tet*(L), *tet*(M), *tetA*(P)	57	*sul1*	82	*tet*(B), *tet*(O), *tetA*(P), *tet*(Q), *tet*(X)	103	*tet*(A), *tet*(M), *tetA*(P), *sul2*, *bla*_TEM-1_		
14	*tet*(O)	41	*tet*(B)	58	*tet*(A), *tet*(M), *tet*(Q), *sul1*	83	*tet*(M), *tet*(O)	105	*tet*(O)		
16	*tet*(M), *tet*(O), *tetA*(P), *tet*(Q), *tet*(X), *sul1*, *bla*_TEM-1_	43	*tetA*(P)	59	*tet*(L), *tet*(M), *tetA*(P)	85	*tet*(M)	106	*sul2*		
17	*tet*(M), *tet*(S)	44	*tet*(M), *tetA*(P)	60	*tet*(M), *tet*(O)	86	*tet*(M), *tet*(S)	108	*tet*(L), *tet*(M), *tet*(X), *sul2*		
18	*tet*(M), *tet*(O), *tet*(S)	45	*tetA*(P)	61	*tetA*(P)	89	*tet*(A)	109	*tet*(O), *tet*(X)		
27	*tetA*(P), *sul1*	46	*tet*(O), *tetA*(P)	62	*tet*(M), *tet*(O), *tetA*(P)	90	*tet*(L), *tet*(M), *tet*(O)	110	*tet*(M), *tet*(O), *tetA*(P), *bla*_TEM-1_		

## Data Availability

Data are contained within the article.

## References

[1] Baros Jorquera C., Moreno-Switt A.I., Sallaberry-Pincheira N., Munita J.M., Flores Navarro C., Tardone R., González-Rocha G., Singer R.S., Bueno I. (2021). Antimicrobial resistance in wildlife and in the built environment in a wildlife rehabilitation center. One Health.

[2] Wright G.D. (2019). Environmental and clinical antibiotic resistomes, same only different. Curr. Opin. Microbiol..

[3] Ramey A.M. (2021). Antimicrobial resistance: Wildlife as indicators of anthropogenic environmental contamination across space and through time. Curr. Biol..

[4] Ventola C.L. (2015). The antibiotic resistance crisis: Part 1: Causes and threats. Pharm. Ther..

[5] Plaza-Rodríguez C., Alt K., Grobbel M., Hammerl J.A., Irrgang A., Szabo I., Stingl K., Schuh E., Wiehle L., Pfefferkorn B. (2021). Wildlife as sentinels of antimicrobial resistance in Germany?. Front. Vet. Sci..

[6] Reshamwala H.S., Mahar N., Dirzo R., Habib B. (2021). Successful neighbour: Interactions of the generalist carnivore red fox with dogs, wolves and humans for continued survival in dynamic anthropogenic landscapes. GECCO.

[7] Martinez J.L. (2009). The role of natural environments in the evolution of resistance traits in pathogenic bacteria. Proc. Biol. Sci..

[8] Radhouani H., Igrejas G., Carvalho C., Pinto L., Gonçalves A., Lopez M., Sargo R., Cardoso L., Martinho A., Rego V. (2011). Clonal lineages, antibiotic resistance and virulence factors in vancomycin-resistant enterococci isolated from fecal samples of red foxes (*Vulpes vulpes*). J. Wildl. Dis..

[9] Carson M., Meredith A.L., Shaw D.J., Giotis E.S., Lloyd D.H., Loeffler A. (2012). Foxes as a potential wildlife reservoir for mecA-positive staphylococci. Vector Borne Zoonotic Dis..

[10] Botti V., Navillod F.V., Domenis L., Orusa R., Pepe E., Robetto S., Guidetti C. (2013). *Salmonella* spp. and antibiotic-resistant strains in wild mammals and birds in north-western Italy from 2002 to 2010. Vet. It..

[11] Radhouani H., Igrejas G., Gonçalves A., Pacheco R., Monteiro R., Sargo R., Brito F., Torres C., Poeta P. (2013). Antimicrobial resistance and virulence genes in *Escherichia coli* and enterococci from red foxes (*Vulpes vulpes*). Anaerobe.

[12] Mo S.S., Urdahl A.M., Madslien K., Sunde M., Nesse L.L., Slettemeås J.S., Norström M. (2018). What does the fox say? Monitoring antimicrobial resistance in the environment using wild red foxes as an indicator. PLoS ONE.

[13] Skarżyńska M., Leekitcharoenphon P., Hendriksen R.S., Aarestrup F.M., Wasyl D. (2020). A metagenomic glimpse into the gut of wild and domestic animals: Quantification of antimicrobial resistance and more. PLoS ONE.

[14] Dias D., Hipólito D., Figueiredo A., Fonseca C., Caetano T., Mendo S. (2022). Unravelling the diversity and abundance of the red fox (*Vulpes vulpes*) faecal resistome and the phenotypic antibiotic susceptibility of indicator bacteria. Animals.

[15] Terentjeva M., Ķibilds J., Avsejenko J., Cīrulis A., Labecka L., Bērziņš A. (2024). Antimicrobial resistance in *Enterococcus* spp. isolates from red foxes (*Vulpes vulpes*) in Latvia. Antibiotics.

[16] Hahaj-Siembida A., Nowakiewicz A., Korzeniowska-Kowal A., Szecówka K., Trościańczyk A., Zięba P., Kania M.G. (2024). Red foxes (*Vulpes vulpes*) as a specific and underappreciated reservoir of resistant and virulent coagulase-positive *Staphylococcus* spp. strains. Res. Vet. Sci..

[17] Benavides J.A., Salgado-Caxito M., Torres C., Godreuil S. (2024). Public health implications of antimicrobial resistance in wildlife at the One Health interface. Med. Sci. Forum.

[18] Ng L.K., Martin I., Alfa M., Mulvey M. (2001). Multiplex PCR for the detection of tetracycline resistant genes. Mol. Cell Probes.

[19] Sáenz Y., Briñas L., Domínguez E., Ruiz J., Zarazaga M., Vila J., Torres C. (2004). Mechanisms of resistance in multiple-antibiotic-resistant *Escherichia coli* strains of human, animal, and food origins. Antimicrob. Agents Chemother..

[20] Batchelor M., Hopkins K., Threlfall E.J., Clifton-Hadley F.A., Stallwood A.D., Davies R.H., Liebana E. (2005). bla(CTX-M) genes in clinical *Salmonella* isolates recovered from humans in England and Wales from 1992 to 2003. Antimicrob. Agents Chemother..

[21] Jouini A., Vinué L., Slama K.B., Sáenz Y., Klibi N., Hammami S., Boudabous A., Torres C. (2007). Characterization of CTX-M and SHV extended-spectrum beta-lactamases and associated resistance genes in *Escherichia coli* strains of food samples in Tunisia. J. Antimicrob. Chemother..

[22] Liu Y.-Y., Wang Y., Walsh T.R., Yi L.-X., Zhang R., Spencer J., Doi Y., Tian G., Dong B., Huang X. (2016). Emergence of plasmid-mediated colistin resistance mechanism MCR-1 in animals and human beings in China: A microbiological and molecular biological study. Lancet Infect. Dis..

[23] Hoffmann M., Sillero-Zubiri C. (2021). *Vulpes vulpes* (amended version of 2016 assessment). IUCN Red List. Threat. Species.

[24] Kalisinska E., Kot K., Łanocha-Arendarczyk N. (2023). Red fox as a potential bioindicator of metal contamination in a European environment. Chemosphere.

[25] Garcês A., Pires I. (2021). Secrets of the astute red fox (*Vulpes vulpes,* Linnaeus, 1758): An inside-ecosystem secret agent serving One Health. Environments.

[26] Colombi D., Roppa F., Mutinelli F., Zanetti M. (2009). La Volpe. Aspetti ecologici, biologici e gestionali in Friuli Venezia Giulia. Reg. Auton. Friuli Venezia Giulia Udine.

[27] Chiari M., Ferrari N., Giardiello D., Lanfranchi P., Zanoni M., Lavazza A., Alborali L.G. (2014). Isolation and identification of *Salmonella* spp. from red foxes (*Vulpes vulpes*) and badgers (*Meles meles*) in northern Italy. Acta Vet. Scand..

[28] Rubini S., Ravaioli C., Previato S., D’Incau M., Tassinari M., Guidi E., Lupi S., Merialdi G., Bergamini M. (2016). Prevalence of *Salmonella* strains in wild animals from a highly populated area of north-eastern Italy. Ann. Ist. Super. Sanità.

[29] Parisi A., Chiara M., Caffara M., Mion D., Miller W.G., Caruso M., Manzari C., Florio D., Capozzi L., D’Erchia A.M. (2021). *Campylobacter vulpis* sp. nov. isolated from wild red foxes. Syst. Appl. Microbiol..

[30] Gambino D., Vicari D., Vitale M., Schirò G., Mira F., Giglia M., Riccardi A., Gentile A., Giardina S., Carrozzo A. (2021). Study on bacteria isolates and antimicrobial resistance in wildlife in Sicily, southern Italy. Microorganisms.

[31] Bertelloni F., Cagnoli G., Biagini F., Poli A., Bibbiani C., Ebani V.V. (2022). Virulence genes of pathogenic *Escherichia coli* in wild red foxes (*Vulpes vulpes*). Animals.

[32] Gambi L., Ravaioli V., Rossini R., Tranquillo V., Boscarino A., Mattei S., D’incau M., Tosi G., Fiorentini L., Donato A.D. (2022). Prevalence of different *Salmonella enterica* subspecies and serotypes in wild carnivores in Emilia-Romagna Region, Italy. Animals.

[33] Carella E., Romano A., Domenis L., Robetto S., Spedicato R., Guidetti C., Pitti M., Orusa R. (2022). Characterisation of *Yersinia enterocolitica* strains isolated from wildlife in the northwestern Italian Alps. J. Vet. Res..

[34] Ebani V.V., Trebino C., Guardone L., Bertelloni F., Cagnoli G., Nardoni S., Sel E., Wilde E., Poli A., Mancianti F. (2022). Occurrence of bacterial and protozoan pathogens in red foxes (*Vulpes vulpes*) in central Italy. Animals.

[35] Ferrara G., Brocherel G., Falorni B., Gori R., Pagnini U., Montagnaro S. (2023). A retrospective serosurvey of selected pathogens in red foxes (*Vulpes vulpes*) in the Tuscany region, Italy. Acta Vet. Scand..

[36] Wang S., Gao X., Gao Y., Li Y., Cao M., Xi Z., Zhao L., Feng Z. (2017). Tetracycline resistance genes identified from distinct soil environments in China by functional metagenomics. Front. Microbiol..

[37] Roberts M.C. Distribution of Tet Resistance Genes Among Gram-Negative Bacteria. https://faculty.washington.edu/marilynr/tetweb2.pdf.

[38] Roberts M.C. Distribution of Tet Resistance Genes Among Gram-Positive Bacteria, *Mycobacterium*, *Mycoplasma*, *Nocardia*, *Streptomyces* and *Ureaplasma*. https://faculty.washington.edu/marilynr/tetweb3.pdf.

[39] Roberts M.C. (2005). Update on acquired tetracycline resistance genes. FEMS Microbiol. Lett..

[40] Ovung A., Bhattacharyya J. (2021). Sulfonamide drugs: Structure, antibacterial property, toxicity, and biophysical interactions. Biophys. Rev..

[41] Pavelquesi S.L.S., de Oliveira Ferreira A.C.A., Rodrigues A.R.M., de Souza Silva C.M., Orsi D.C., da Silva I.C.R. (2021). Presence of tetracycline and sulfonamide resistance genes in *Salmonella* spp: Literature review. Antibiotics.

[42] Wang N., Yang X., Jiao S., Zhang J., Ye B., Gao S. (2014). Sulfonamide-resistant bacteria and their resistance genes in soils fertilized with manures from Jiangsu Province, southeastern China. PLoS ONE.

[43] Castanheira M., Simner J., Bradford P.J. (2021). Extended-spectrum β-lactamases: An update on their characteristics, epidemiology and detection. JAC-AMR.

[44] Muhammad I., Golparian D., Dillon J.A., Johansson A., Ohnishi M., Sethi S., Chen S.C., Nakayama S., Sundqvist M., Bala M. (2014). Characterisation of blaTEM genes and types of β-lactamase plasmids in *Neisseria gonorrhoeae*—The prevalent and conserved blaTEM–135 has not recently evolved and existed in the Toronto plasmid from the origin. BMC Infect. Dis..

[45] Galhano B.S.P., Ferrari R.G., Panzenhagen P., de Jesus A.C.S., Conte-Junior C.A. (2021). Antimicrobial resistance gene detection methods for bacteria in animal-based foods: A brief review of highlights and advantages. Microorganisms.

[46] Blanco-Peña K., Esperón F., Torres-Mejía A.M., de la Torre A., de la Cruz E., Jiménez-Soto M. (2017). Antimicrobial resistance genes in pigeons from public parks in Costa Rica. Zoonoses Public Health.

[47] Di Francesco A., Renzi M., Borel N., Marti H., Salvatore D. (2020). Detection of tetracycline resistance genes in European hedgehogs (*Erinaceus europaeus*) and crested porcupines (*Hystrix cristata*). J. Wildl. Dis..

[48] Di Francesco A., Salvatore D., Gobbi M., Morandi B. (2023). Antimicrobial resistance genes in a golden jackal (*Canis aureus* L. 1758) from central Italy. Vet. Res. Commun..

[49] Di Francesco A., Salvatore D., Ranucci A., Gobbi M., Morandi B. (2024). Antimicrobial resistance in wildlife: Detection of antimicrobial resistance genes in Apennine wolves (*Canis lupus italicus* Altobello, 1921) from central Italy. Vet. Res. Comm..

[50] Dolejska M., Literak I. (2019). Wildlife is overlooked in the epidemiology of medically important antibiotic-resistant bacteria. Antimicrob. Agents Chemother..

[51] Zeballos-Gross D., Rojas-Sereno Z., Salgado-Caxito M., Poeta P., Torres C., Benavides J.A. (2021). The role of gulls as reservoirs of antibiotic resistance in aquatic environments: A scoping review. Front. Microbiol..

